# From real to virtual prism adaptation therapy: a systematic review on benefits and challenges of a new potential rehabilitation approach

**DOI:** 10.3389/fpsyg.2024.1391711

**Published:** 2024-06-20

**Authors:** Laura Culicetto, Andreina Giustiniani, Viviana Lo Buono, Valentina Cazzato, Alessandra Falzone, Carmelo Mario Vicario, Angelo Quartarone, Silvia Marino

**Affiliations:** ^1^IRCCS Centro Neurolesi Bonino Pulejo, Messina, Italy; ^2^Dipartimento di Scienze Cognitive, Psicologiche, Pedagogiche e Degli Studi Culturali, Università di Messina, Messina, Italy; ^3^School of Psychology, Faculty of Health, Liverpool John Moores University, Liverpool, United Kingdom

**Keywords:** virtual reality, prismatic adaptation, virtual prism adaptation therapy, spatial neglect, rehabilitation Italiano (Italia)

## Abstract

Prism adaptation (PA) is a sensorimotor technique that has been shown to alleviate neglect symptoms. Due to its demonstrated functional effectiveness, PA has recently been implemented in virtual reality environments. However, research on virtual prism adaptation (VPA) is limited and it lacks a standardized methodological approach. It is crucial to investigate whether VPA can be effective in inducing traditional effect of PA and to have potential utility in a rehabilitation context. Clarifying this aspect would allow the use of VPA in a wider range of contexts and neurological disorders, with the additional opportunity to overcome PA traditional limits. The aim of the present study is to revise current literature on VPA in both healthy individuals and patients highlighting also its advantages and limitations. Studies performed between 2013 and 2023 and fulfilling the inclusion criteria were searched on three electronic databases, by combining the terms “Virtual prism adaptation” and “Virtual prism adaptation therapy. Out of 123 articles, only 16 met the inclusion criteria. The current literature review suggests that VPA may serve as a potentially useful tool for inducing visuomotor adaptation, with most studies conducted in healthy individuals. The high variability in the methodologies observed among studies suggests that more standardized approaches are needed to gain a deeper understanding of the mechanisms underlying adaptation and aftereffects when PA is administered in a virtual environment. Future studies should also address practical applications and clinical efficacy of VPA, particularly in patients with spatial neglect.

## 1 Introduction

Prismatic adaptation (PA) is a non-invasive technique used in the rehabilitation of patients with unilateral spatial neglect (USN), a prevalent and complex sensorimotor disorder that occurs post-stroke. USN is characterized by deficits in attention and awareness toward the side of space opposite to the brain damage (Vallar et al., 2003). Given the clinical significance of USN, it is essential to explore the efficacy of innovative therapeutic interventions such as prism adaptation (PA). Introduced by Rossetti et al. (1998), PA has emerged as a promising bottom-up approach to alleviate USN symptoms. This intervention entails wearing prism goggles that laterally shift the visual field, coupled with the use of a device such as a horizontal board with target dots (Schintu et al., 2014) or a box (Magnani et al., 2011), for pointing movements. Patients are instructed to point toward targets using their right index finger. PA temporarily alters sensorimotor mapping by shifting vision laterally (Rossetti et al., 1998) while participants are engaged in a pointing task toward visual targets. Initially, movements typically exhibit errors in the direction of the prismatic shift, but these errors diminish after repeated attempts, indicating adaptation. Following prism removal, aftereffects (AEs) often manifest as errors in the opposite direction (Redding and Wallace, 2002). Factors influencing AE include the degree of lateral shift (Gammeri et al., 2020) and awareness of the visual shift. Interestingly, the alteration of motor movements experienced during PA persists after the removal of the prisms, with sensorimotor and visuospatial effects lasting at least 35 min (Schintu et al., 2014).

Two main neural mechanisms have been proposed to account for PA effects: recalibration and realignment (Redding and Wallace, 2006). Recalibration is a compensatory mechanism that modifies motor commands while individuals carry actions such as reaching objects; it involves a rapid adjustment of the motor plan to minimize terminal errors. Realignment, on the other hand, is a slower, spontaneous process that can reconfigure the sensory maps perturbed by prism shift, resulting in a correction of motor strategy (Prablanc et al., 2020).

Several studies have reported the beneficial effects of PA on various populations including healthy individuals (Bonaventura et al., 2020; Turriziani et al., 2021; Magnani et al., 2022), psychiatric populations (Magnani et al., 2022), and neurological populations, particularly those suffering from USN (Shiraishi et al., 2008; Serino et al., 2009) characterized by an improvement in daily activities such as reading, writing, and wheelchair navigation (Dijkerman et al., 2003; Angeli et al., 2004a,b; Rode et al., 2006; Jacquin-Courtois et al., 2008; Watanabe and Amimoto, 2010). Furthermore, PA based-rehabilitation has been shown to reduce deficits in various domains, including neglect (Rossetti et al., 1998; Làdavas et al., 2001; Dijkerman et al., 2003; Maravita et al., 2003; Jacquin-Courtois et al., 2010), visuospatial (Farnè, 2002) and postural deficits (Tilikete et al., 2001; Hugues et al., 2015).

Interestingly, the sensorimotor AEs of PA extend to cognitive domains, including mental imagery tasks that do not involve manual responses or overt visual scanning (Rode et al., 1999; Rossetti et al., 2004; Aiello et al., 2013). These AEs are influenced by the direction of visual displacement. For instance, while right-deviating PA improves neglect symptoms, and left-deviating PA is ineffective in neglect patients; however, healthy individuals experience significant cognitive changes after left-deviating, but not right-deviating PA.

Due to the enormous potential of PA therapy, recent studies have attempted to optimize PA by introducing a novel virtual reality-based PA protocol. Specifically, when implemented in VR, PA follows the same procedure as the traditional approach, but the pointing task may be executed with or without touch controllers while wearing virtual reality headsets.

With the increasing accessibility and widespread use of virtual reality (VR) systems, new opportunities for rehabilitation and psychotherapy support have emerged (Glize et al., 2017; Maggio et al., 2019; Leonardi et al., 2021; Lucifora et al., 2022; Maresca et al., 2022; Vicario and Martino, 2022; De Luca et al., 2023; Formica et al., 2023; Vicario et al., 2023a,b). VR is defined as interactive simulations created by computer hardware and software that immerse users in environments closely emulating real-world objects and events (Weiss et al., 2006) gaining popularity in rehabilitation settings for treating motor and cognitive disorders (Tieri et al., 2018). Three types of VR device can be identified based on immersion levels (Bamodu and Ye, 2013): non-immersive (NIVR), semi-immersive (SIVR), and immersive (IVR). NIVR, which utilizes 2D interfaces (such as mouse and keyboard or gamepad/joysticks) along with computer or console gaming systems, partially immerses users in the virtual environment (VE) while allowing them to remain aware of the outside world (Shahrbanian et al., 2012). SIVR involves the use of a large screen for VE projection and advanced interface devices such as cyber gloves, haptic feedback devices, or infrared cameras, enabling interaction with the VE while perceiving the real world (Bamodu and Ye, 2013). IVR, featuring a head-mounted display and 3D input devices, fully immerses users in the VE (Shahrbanian et al., 2012). VR offers a unique opportunity to modulate multiple parameters, enhancing the power of virtual prisms and isolating patients from external stimuli.

This new rehabilitation approach has the potential to address some limitations of PA. It can induce different degrees of deviation using the same setup and alleviate the traditional difficulty associated with moving the panel, especially for hospitalized patients. Furthermore, virtual PA (VPA) allows for a more precise digital quantification of both errors during PA and the occurrence of AEs.

Due to the high flexibility and personalization offered by the VPA, understanding its effectiveness will have important research and clinical implications, as well as improving rehabilitation outcomes. Therefore, here we conducted a systematic review of the current literature focusing on the use of VPA in healthy individuals and patients. The results of this review may pave the way to novel rehabilitation approaches for patients with different neurological conditions, including neglect.

## 2 Methods

A systematic review was conducted to investigate the efficacy of VPA. Although this review was not registered in a specific database, it was conducted in accordance with the Preferred Reporting Items for Systematic Reviews and Meta-Analyses (PRISMA) guidelines (Moher, 2009; Page et al., 2021).

### 2.1 PICOS model

We employed the PICOS (Population, Intervention, Comparison, Outcome and Study design) model to shape our research question (Page et al., 2021). Our target population include adults (>18 years) affected by USN or healthy individuals. The intervention examined was VPA, administered using various VR systems. The intervention involved visual-motor tasks that included adjustments to visual feedback through virtual prisms, aiming to induce sensorimotor adaptation.

Comparisons were typically made between different degrees of virtual lenses, or with physical prism glasses with or without deviation. The main outcomes assessed were the magnitude and duration of visuomotor adaptation effects, specifically the aftereffects observed following the removal of the prisms and improvements in test performance. The study design included both randomized controlled trials (RCTs) and non-randomized studies to ensure a comprehensive overview of the current understanding of VPA in the field.

### 2.2 Search strategy

Considering that this is a new technology, the most recent search for articles published between 2013 and 2023 was conducted in April 2024 across three databases: Scopus, PubMed, and Web of Science. The following query was entered in each of the chosen database: (“virtual reality” OR “virtual environment” OR “immersive environment” OR “simulated environment” OR “artificial environment”) AND (“prismatic adaptation” OR “prism adaptation” OR “prismatic goggles” OR “prism goggles” OR “prism therapy” OR “prism-induced adaptation” OR “virtual prism adaptation” OR “virtual prism adaptation therapy”).

We considered articles that focused on advantages and limits of VPA and application on both healthy subjects and patients. We explored the implementation of VPA in diverse clinical and experimental settings, examining how it addresses the limitations of traditional PA.

### 2.3 Study selection

Articles screening based on titles, abstracts, and full texts, was performed independently by two investigators (L.C. and A.G.). Any disagreements on the inclusion and exclusion criteria were resolved by involving a third researcher (V.L.B.). The list of articles was then refined for relevance, revised, and summarized, with key topics identified based the inclusion/exclusion criteria.

### 2.4 Inclusion/exclusion criteria

Studies were included if they met the following criteria: (1) were original articles, either randomized controlled studies (RCT) or not; (2) they investigated VPA in patients with neurological diseases or healthy individuals; (3) the effects of VPA were reported in terms of single session or rehabilitation outcomes and (4) they were written in English.

Studies were excluded if they met any of the following criteria: full-text unavailability, conference abstracts, articles not written in English, and qualitative studies.

### 2.5 Data extraction

Following the full-text selection, data were extracted from the included studies and reported in a table using Microsoft Excel (Version 2021). The extracted data included: study title, first author name, year of publication, study aims and design, sample size, type of participants, type of intervention and control, baseline performance, type of outcome and time-points for assessment, results, and key conclusions. Moreover, the agreement between the two reviewers (L.C. and A.G.) was assessed using the kappa statistic. The kappa score, with an accepted threshold for substantial agreement set at >0.61, was interpreted to reflect excellent concordance between the reviewers. This criterion ensures a robust evaluation of the inter-rater reliability, emphasizing the achievement of a substantial level of agreement in the data extraction process.

### 2.6 Risk of bias assessment

The risk of bias was assessed using the Cochrane tool for non-randomized controlled trials (ROBINS-E) (Bero et al., 2018) which comprises seven domains: (i) Bias due to confounding; (ii) Bias arising from measurement of the exposure; (iii) Bias in selection of participants into the study; (iv) Bias due to post-exposure interventions; (v) Bias due to missing data; (vi) Bias arising from measurement of the outcome; (vii) Bias in selection of the reported result.

Results of risk of bias assessments were visualized using the Cochrane risk-of-bias visualization tool.

The risk of bias in randomized controlled studies (RCT) was assessed using the revised Cochrane risk of bias (RoB 2) tool (Sterne et al., 2019) which comprises five domains: (i) bias arising from the randomization process, (ii) bias due to deviations from the intended intervention, (iii) bias due to missing outcome data, (iv) bias in the measurement of the outcome, (v) bias in the selection of the reported result.

## 3 Results

### 3.1 Synthesis of evidence

**Figure 1:**
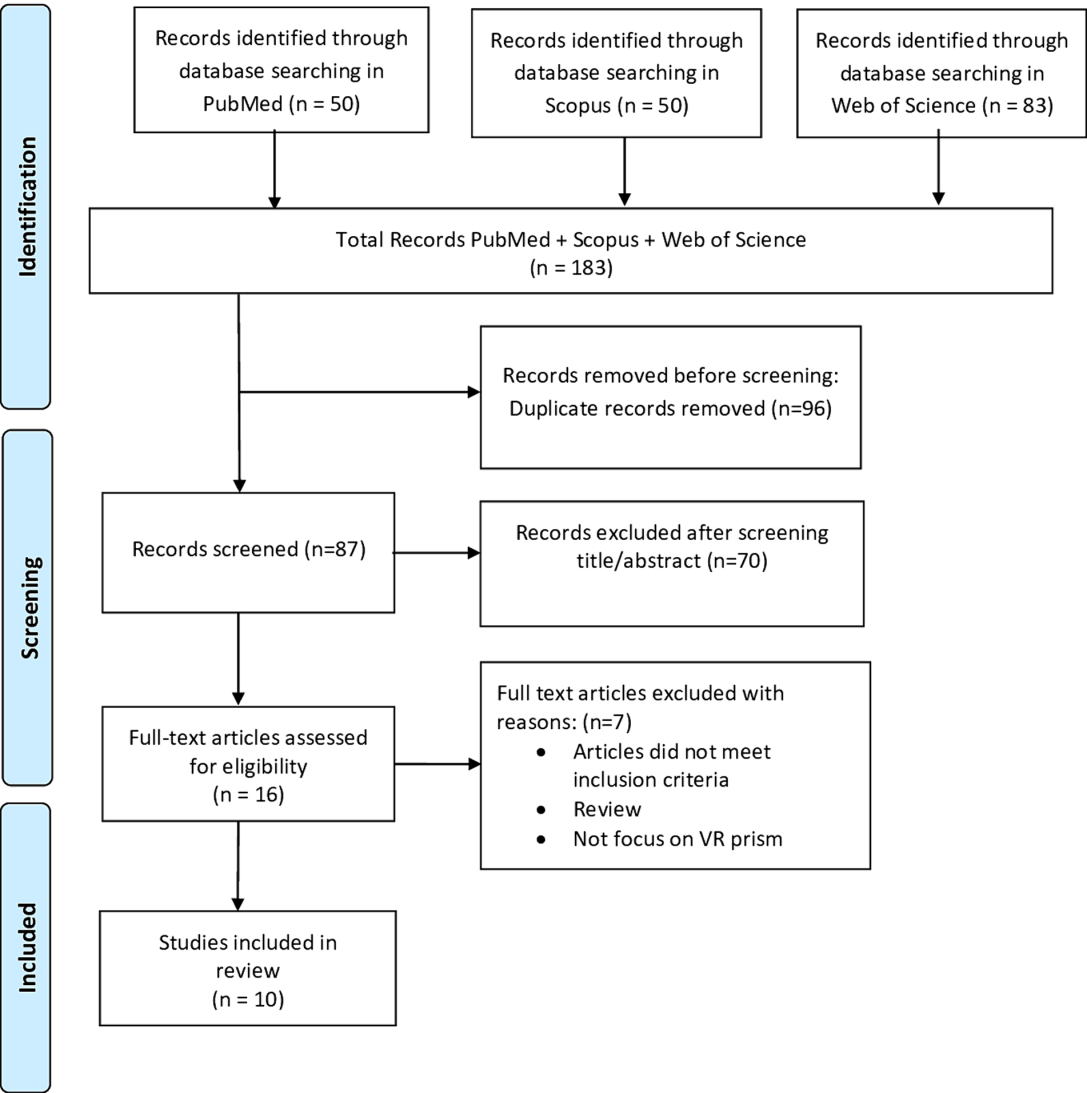
PRISMA flowchart showing identification, inclusion, and exclusion of studies in the systematic review.

Out of the 123 articles initially identified, 48 duplicates were removed; following title and abstract screening 43 items were excluded; 32 studies underwent full-article screening to assess eligibility. Finally, 16 studies were included in the review as they met the inclusion criteria (see Figure 1).

### 3.2 Key findings from included studies

The majority of the studies, specifically fourteen, included in the present review were conducted on healthy participants. Indeed, we found only two studies focusing on patients with neglect (Table 1). Carter et al. (2016) explored whether visuomotor adaptation (VMA) can be induced in neurologically intact individuals through a VR game that alters the integration between motor actions and visual feedback. Their study introduces a novel approach to VMA using VR in a low-immersion setting, expanding the potential for therapeutic use. The application of Microsoft Kinect v2 sensor demonstrates the feasibility of using commercially available VR components in both research and therapy (Carter et al., 2016). Fuji et al. (2018) developed a VR-based rehabilitation support system for USN, observing that prism adaptation effects seem achievable in healthy subjects, suggesting a need for replication with patients. Kim et al. (2017) conducted a preliminary study on VPA therapy using immersive VR, finding similar effects during the adaptation and post-adaptation phases to conventional prism therapy. Cho et al. (2020) initially validated a virtual prismatic adaptation therapy (VPAT), exploring whether translated hand movements in VR could induce angle overshooting and behavioral adaptation similar to traditional PA. The results showed pointing errors by the VPAT system comparable to the conventional PA. Interestingly, a subsequent study (Cho et al., 2022) combining VPAT with functional near infrared spectroscopy (fNIRS) demonstrated that the observed results in the pointing task during VPAT are paralleled by an activation of the right dorsolateral prefrontal cortex and the frontal eye field. However, this study did not assess the transfer to visuo-motor cognitive domains.

**Table 1:** Main characteristics of the included studies.

Study	Study design	Sample	Prisms deviation (degrees)	Intervention	VR system	Comparison	Outcome measures	Results
Kim et al. (2017)	Validation study	4 HP	10°, 20°	VR [Non-prism phase, prism exposure and non-prism (post-adaptation)]	Oculus Rift DK2 and Leap Motion controller	No control	Pointing error	Rightward deviation during prism and leftward deviation in the post adaptation
Bourgeois et al. (2022)	RCT double blind study	15 neglect patients; 5 females (age range = 46–75).	15°, 30°	VR	Vive VR headset	0° deviation	OLP; Line bisection; Item cancelation	Presence of robust OLP; no effects in line bisection and item cancelation
Cho et al. (2022)	Feasibility study	14 HP; 7 females (27.8 ± 4.1)	10°,20°	VR: 4 sequential phases: pre-VPAT, VPAT-10°, VPAT-20°, post-VPAT	Oculus Rift DK2 and Leap Motion controller + fNIRS	No control	Pointing error and brain activation	AE as in conventional PA; Activation in the rDLPFC and the FEF
Ramos et al. (2019)	Cross-over study	20 HP, 13 females (25.9 ± 5.5) 7 males (28.3 ± 3.1)	10°	VRR and VRS	HTC VIVE VR headset	PCP	Pointing error	AE in VRR larger than in PCP
Gammeri et al. (2020)	Single-blind dose response study	48 HP, 34 females (22.8 ± 3.3)	10°, 20°, 30°	VR: Before adaptation, adaptation, after adaptation, and recalibration	Vive VR system	No deviation	Bisection and landmark tasks	Changes in the bisection task only with 30° of deviation
Faity et al. (2023)	Validation study	10 HP; 7 females (23 ± 39)	10°	VR: 2 blocks [exposure and recalibration (2 h after exposure)] including three conditions: mSSA, vSSA and VOL	HTC Vive	Real PA	Pointing error and presence of AE	AE was similar in VR and in real PA but not present in the recalibration phase.
Ishida and Higa (2023)	Validation study	13 HP	Na	VR (HaA and HeA)	Oculus Quest2 as a VR headset.	No control	Posture and head position	Only HeA induces changes in the visual field and in posture
Cho et al. (2020)	Validation study	4 HP; 3 females (age range:18–50)	10°, 20°	4 sequential phases: pre-VPAT, VPAT-10°, VPAT-20°, post-VPAT	VR HDM + fNIRS	No control	Pointing error	Pointing errors for each participant in the VPAT similar to those reported by the literature for real PA
Carter et al. (2016)	Proof-of-principle study	7 HP; 6 females (ages 23–27 years)	Na	VR inducing a virtual shift: 3 phases: pre-adaptation, adaptation, and de-adaptation	VR system (Kinect v2 gaming sensor coupled with online games)	Optical shift induced by real googles	Reaching task	VR induces a shift in AE similar to that of the real googles
Fuji et al. (2018)	Feasibility study	6 HP (approximately 20 years old)	20 diopters	VR	Oculus Rift and Oculus Touch.	No control	Adaptation and reaching task	Adaptation in the VR is similar to that reported by the current literature for real PA.
Bourgeois et al. (2021)	RCT	48 HP; 27 females (18–46 years)	30°	3 phases (pre-tests, adaptation, and post-tests)	VR system	0° deviation	Line bisection and landmark tasks	Visual but not auditory verbal- feedback induces AE following PA
Chen et al. (2022)	Case-series study	3 stroke survivors patients	Na	VR treatment game required reaching and touching the nose of an animal	HTC Vive	No control	BIT-c	Improvement of USN after multi session treatment
Wilf et al. (2023)	RCT	45 HP (23 ± 3; 23 ± 4)	20°	2 phases of VR training with 2 phases of fMRI	Oculus Rift and Oculus Touch	Sham VRPA	Functional connectivity MRI to assess brain activity patterns before and after VRPA training	A brief VRPA exposure can change cortical connectivity
Wähnert and Gerhards (2022)	RCT	30 HP, 16 female (aged 22–29)	20 dioptres	4 phases: familiarization, baseline, adaptation, and readaptation phase	HTC Vive	No pointing movements and no visual displacement	Magnitude and persistence of AE	5 pointing movements in VR are sufficient to elicit AE
Wilf et al. (2021)	RCT	60 HP, 28 female (25 ± 4)	25° visual shift	adaptation training stage using a virtual reality and haptic robotic setup	Oculus Rift	Sham VRPA	Pointing error, landmark task	VPA induces effects generalizing to untrained portion of space, even for robot-guided movements during adaptation
Ishida and Higa (2024)	Validation study	15 HP	Shift angle −15°	hand and head position adaptation tasks	Oculus Quest 2	No control	The adaptability of shifts (θpos) and estimation of the subject’s posture during task performance (θor)	The head position adaptation task could generate PA in the upper trunk system

HP, healthy participants; VPAT, virtual prism adaptation therapy; OPL, open loop pointing; AE, after effect; rDLPFC, right dorsolateral prefrontal cortex; FEF, frontal eye field; VRR, virtually simulated prism-deviation using virtual reality (ROTATE); VRS, virtually simulated prism-deviation using virtual reality (SKEW); PCP, prism-deviation using a standard set of prism goggles and PC; Na, Not available; fNIRS, functional near-infrared spectroscopy; HaA, hand adaptation; HeA, head adaptation; mSSA, straight ahead task; vSSA, virtual subjective straight ahead; BIT-c, behavioral inattention test; USN, unilateral spatial neglect; fMRI, functional magnetic resonance imaging; RCT, Randomized controlled trial; VRPA, virtual reality prismatic adaptation.

The integration of VR with tools like fNIRS in studies by Wilf et al. (2023) showed promising results, demonstrating that VR could effectively modify large-scale cortical connectivity and influence the processing of naturalistic stimuli.

Bourgeois et al. (2021) found that VR provides a flexible environment for introducing mismatches between visual and proprioceptive inputs, facilitating a gradual adaptation process that could be imperceptible to the user, hence enhancing the effectiveness of the therapy. The gradual introduction of mismatches, as well as the controlled environment VR offers, allow for precise management of visual displacement and interaction number, creating an adjustable setup for sensorimotor adaptation therapy. Expanding on the theme of sensorimotor adaptation, another study (Wähnert and Gerhards, 2022) verifies that engaging in thirty-five pointing actions enhances both the strength and the longevity of the aftereffect corroborating results from studies on PA (Dewar, 1970; Fernández-Ruiz and Díaz, 1999). Authors have stated that the use of VR technology allows for precise control over the visual displacement and the number of interactions, offering a flexible and easily adjustable setup for sensorimotor adaptation therapy.

A single study (Ramos et al., 2019) involving healthy subjects compared the effects of rotating (ROTATE-VRR) or skewing (SKEW-VRS) the visual field in VR with the effects of real PA and showed that both the ROTATE and the SKEW conditions produced larger prismatic after-effects than traditional prism goggles. On the other hand, Gammeri et al. (2020) explored the role of angles deviation (i.e., 10-, 20-, 30-degrees) in inducing the transfer of PA effects to a landmark and a bisection task, respectively. The authors reported that robust PA effects may only be induced by large deviations (i.e., 30-degrees) (Gammeri et al., 2020). Kim et al. (2017) and Cho et al. (2020) developed a virtual prism adaptation therapy combining immersive VR with a depth-sense camera and tested the effects of this system in healthy individuals with the aim to overcome limits of traditional PA. These studies report similar effects for VR and traditional PA, thus highlighting the potential benefit of a mobile device, especially for post-hospital use. Similarly, Ishida and Higa (2023, 2024) developed a virtual prism adaptation system including posture measurement functionality, expanding the application range of prism adaptation to the whole body.

Faity et al. (2023) used a comparative approach to evaluate real and virtual prism exposures and noted that virtual prisms are as effective as physical prisms in inducing aftereffects; however, these effects did not persist beyond 2 h, regardless of the exposure modality. Furthermore, high correlation between the angles of deviation obtained with the HTC Vive® and Zebris systems, suggests the reliability of the HTC Vive® for measuring deviation angles. In a double-blind study, Bourgeois et al. (2022) rehabilitated neglect patients with VPA at varying degrees (0°, 15°, or 30°) and measured their performance in line bisection and item cancelation tasks before and after adaptation. Despite the robustness of the open loop pointing effect, no improvements in test performance were observed. Moreover, to assess the potential benefits of VPA on spatial neglect, a multi-baseline experiment was conducted involving three individuals with chronic left-sided neglect following stroke (Chen et al., 2022). All participants showed enhanced spatial neglect immediately after undergoing five sessions. In another study, Wilf et al. (2021) reported that the strong point of VPA is that it induced and generalized aftereffects in non-trained areas of space, even for robot-guided movements during adaptation. Moreover, the ecological and immersive setup, along with the haptic robotic device, is highlighted as a promising tool for treating neglect patients, with and without motor impairment.

### 3.3 Risk of bias

**Figure 2:**
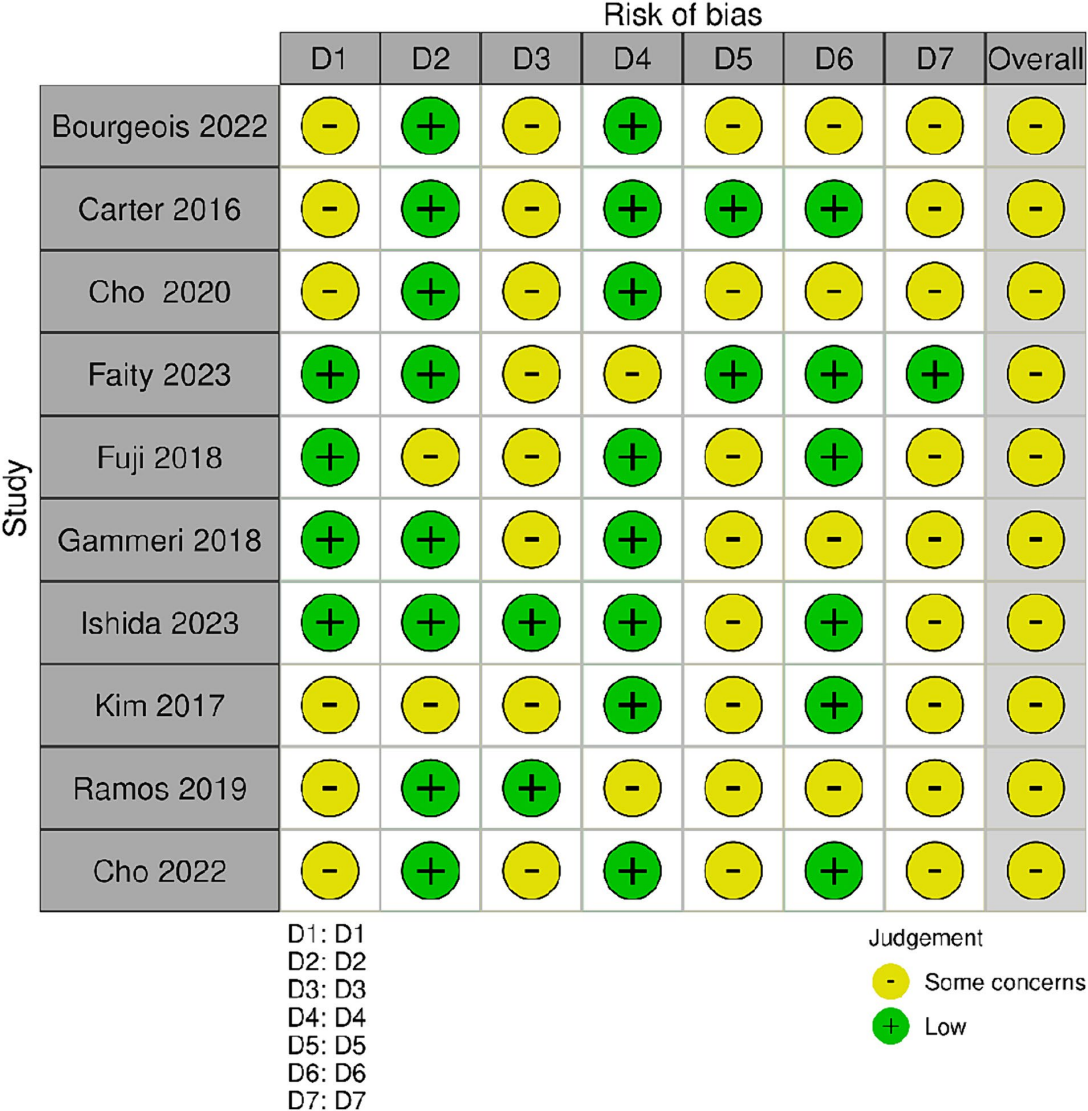
Risk of bias summary. Review author’s judgments about each risk of bias item for the included studies. Bias due to confounding (D1); bias arising from measurement of the exposure (D2); bias in selection of participants in to the study (D3); bias due to post-exposure interventions (D4); bias due to missing data (D5); bias arising from measurement of the outcome (D6); bias in selection of the reported result (D7).

**Figure 3:**
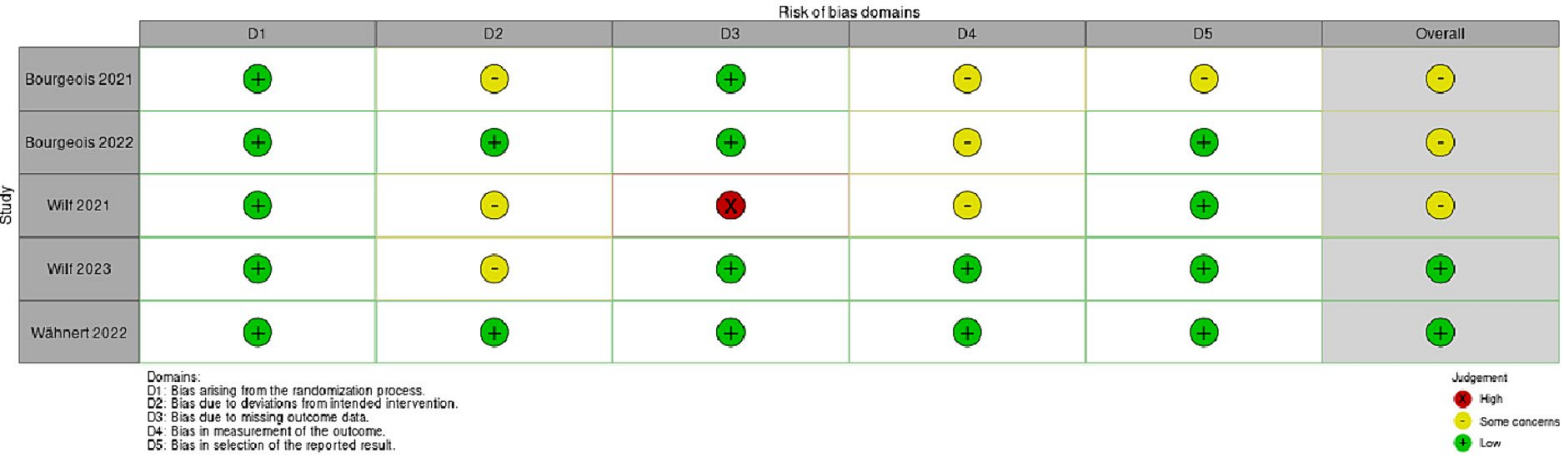
This figure shows the risk of bias (RoB 2) randomized controlled studies (RCT) regarding virtual prism adaption (VPA).

The Cochrane Risk of Bias Assessment Tool (ROBIN-E) (Bero et al., 2018) was used to assess the risk of bias of the articles included in this review. Figure 2 shows the summary of the risk of bias assessment, while the graph depicts the distribution of bias concerns across the included studies. Five studies (Carter et al., 2016; Kim et al., 2017; Ramos et al., 2019; Cho et al., 2020, 2022) raised concerns about bias due to confounding factors. Additionally, some studies exhibited concerns related to bias from the measurement of exposure and post-exposure interventions. Specifically Fuji et al. (2018) and Kim et al. (2017) for exposure measurement, Faity et al. (2023), Ramos et al. (2019), and Ishida and Higa (2024) for post-exposure interventions. Moreover, all studies selected except four, Ishida and Higa (2023) and Ramos et al. (2019), Chen et al. (2022), and Ishida and Higa (2024), showed a low risk of bias in the selection of participants. Conversely, Carter et al. (2016), Faity et al. (2023), and Chen et al. (2022) displayed lower bias due to missing data. Bias arising from the measurement of the outcome was a concern in three studies (Ramos et al., 2019; Cho et al., 2020; Gammeri et al., 2020; Bourgeois et al., 2022). Finally, all studies except one (Faity et al., 2023) had concerns regarding the selection of the reported results (Figure 3).

The risk of bias in randomized controlled studies (RCT) was assessed using the revised Cochrane risk of bias (RoB 2) tool (Sterne et al., 2019). All five studies report low risk of bias arising from the randomization process (Bourgeois et al., 2021; Wilf et al., 2021; Bourgeois et al., 2022; Wähnert and Gerhards, 2022; Wilf et al., 2023). Only three studies show some concerns about the risk of bias due to deviations from intended intervention (Bourgeois et al., 2021; Wilf et al., 2021, 2023). A high risk of bias due to missing outcome data was reported in the study conducted by Wilf et al. (2021). Bourgeois et al. (2021, 2022) studies displayed some concerns about bias in measurement of the outcome. Finally, only the study by Bourgeois et al. (2021) showed some concerns about bias in selection of the reported result.

## 4 Discussion

The results of this systematic review suggest the potentiality of VPA in inducing visuomotor adaptation in healthy subjects, comparable to real PA. While research in this field is still in its early stages and only preliminary conclusions can be drawn, there is a growing interest in exploring the potential implementation of VR based PA therapy (Cho et al., 2020; Gammeri et al., 2020). It is noteworthy that, although the primary aim of the current study was not to directly compare traditional PA effects with VPA, many of the included studies examining VPA compared its effects to those of traditional PA. For instance, Ramos et al. (2019) compared prism AE in the real world after the removal of prism goggles, and the VR AE within the VE while wearing VR goggles, reporting larger prismatic AEs for VPA. Similarly, a recent study examining AE in VPA (Faity et al., 2023) found comparable AE similar to those observed with traditional PA (Faity et al., 2023). Interestingly, like traditional PA, these effects have been shown to vary across different cognitive tasks (Gammeri et al., 2020). However, this study highlights that the relationship between prism deviation and transfer effects is complex (Gammeri et al., 2020). Transfer effects were only evident with larger prismatic deviations (around 30 degrees) while they were absent with smaller deviations (20 degrees or less). This observation raises questions about the optimal degree of prismatic deviation for effective VPA and suggests that a one-size-fits-all approach may not be suitable.

An important limitation we found in the current literature is the lack of research involving patients. Specifically, we identified only two studies investigating the effect of VPA in patients with neglect (Bourgeois et al., 2022; Chen et al., 2022) which reported robust VPA effects on open-loop pointing. Unfortunately, in the study by Bourgeois et al. (2022), this effect did not translate into a significant improvement in clinical assessments, including cognitive tasks. The authors suggested that several factors might explain the lack of effect, including the small sample size and type of pointing task utilized in the study. Similar inconsistent results have also been reported for patients with neglect undergoing traditional PA (Morris et al., 2004). These discrepancies in findings have previously raised concerns about the effectiveness of PA as a rehabilitation method for this and other clinical populations. On the contrary, Chen et al. (2022) showed improvements as measured by BIT-c accuracy, but not in terms of laterality in patients with USN after VPA training. These findings contrast with those of Bourgeois et al. (2022) who reported no change in spatial bias or improvement in USN in any condition. However, there are substantial differences between the two studies that may have affected the results. In Bourgeois et al. (2022) participants utilized a handheld controller for responses, while in Chen’s study, participants used their hands directly. The VR program described by Bourgeois et al. (2022) may have lacked a game-like quality compared to Chen et al.’s (2022) program. Another significant difference lies in the assessment methods as Bourgeois et al. (2022) assessed participants within the VR headset environment, whereas Chen et al. (2022) evaluated participants using traditional 2-D tests outside the virtual environment. However, it is important to consider differences in methodology when comparing results across studies. Future research should aim to standardize experimental procedures for VPA application to identify the most effective PA approach, whether in virtual or physical reality, to maximize benefits for patients. Based on the evidence collected, we propose that an effective VPA approach should include precise visual deviations tailored to individual needs, customizable protocols that adapt in real-time, integration of multisensory feedback, and extended repetitive practices to enhance therapeutic outcomes. We emphasize the importance of developing standardized VPA protocols that incorporate these elements to maximize rehabilitation efficacy in virtual reality settings. Indeed, a recent study utilized fNIRS (Bero et al., 2018) to examine brain activity during VPA, revealing a significant activity in the right frontal eye field and in the dorsolateral prefrontal cortex (DLPFC) during and following VPAT. This result aligns with previous literature on traditional PA, which has consistently reported activation in a broad network including the frontal lobes, the parietal cortex and cerebellum as assessed with functional magnetic resonance imaging (fMRI) (Luauté et al., 2009; Chapman et al., 2010; Küper et al., 2014; Panico et al., 2020). Future MRI studies should further explore whether the same network involved in traditional PA is also implicated in VPA.

The selection of VR hardware and tracking systems is also critical in VPA research. High-cost, space-consuming PC-connected VR system (Kim et al., 2017; Cho et al., 2020) may limit the accessibility of VPA interventions. To address these challenges, Ishida and Higa (2023) recently proposed a novel VPA system designed to create a rehabilitation space (for instance for USN) suitable for use in both hospital and home setting.

This system may expand the use of prism adaptation to involve full-body participation, enhancing the therapeutic process by integrating postural feedback and encouraging spontaneous head orientation toward the neglected side (Ishida and Higa, 2023). The development of the VR system opens new perspectives for applying PA to a collection of multiple motor units, such as the upper trunk system, and highlights the potential of this approach for postural correction in USN patients, along with possible applications in detecting cerebellar disorders (Ishida and Higa, 2024). Future studies are needed to explore the applicability and effectiveness of this system. Regarding the methodologies and devices used in the studies reviewed, such as Oculus Rift and Oculus Quest, they present challenges in synthesizing a comprehensive understanding of VPA’s efficacy. Different VR devices vary in display resolution, field of view, and tracking capabilities, impacting user experience and the effectiveness of VPA studies. Higher resolution and better tracking can improve visual feedback, crucial for prism adaptation. The comfort and usability of VR headsets also affect participation and outcomes, as heavier or less ergonomic headsets can cause fatigue, limiting engagement time. Furthermore, variability in device calibration and customization can influence how users perceive and adapt to virtual environments, potentially skewing study results. For example, the Quest 2 offers a cable-free experience with a built-in battery and a higher-resolution, higher-refresh rate and brighter display compared to the Rift, which requires connection to a PC via DisplayPort and USB (Jost et al., 2021; Carnevale et al., 2022). Therefore, the selection of devices should be carefully considered when planning VPA studies.

Additionally, the current literature highlights some disadvantages associated with the use of VPA. For example, actual VR systems may cause discomfort or head pain thus affecting user experience (Cho et al., 2020). Moreover, the occurrence of motion sickness, a common side effect associated with immersive VR or head-mounted devices, is an aspect requiring careful consideration in the use of VPA (Munafo et al., 2017). Furthermore, participants may encounter challenges in performance due to the weight and design of the headset, potentially affecting user comfort and compliance during longer sessions (Ramos et al., 2019; Cho et al., 2020). However, these discomforts can be alleviated by strategic configuration of the virtual environment (Cho et al., 2020).

On the other hand, the current literature highlights several key advantages of VPA, particularly its ability to offer precise control over the degree of optical deviation, a challenge often encountered with traditional wedge prisms (Bourgeois et al., 2022). This precision is crucial for accurately tracking and quantifying pointing errors throughout therapy, a feature that can be pivotal for establishing effective home-based rehabilitation programs (Cho et al., 2022). In experimental setup, VR provides a more effective blinding of participants if compared with traditional PA (Gammeri et al., 2020). The removal of extraneous visual stimuli enabled by VPA ensures a controlled environment for evaluating adaptation effects. In other words, the immersive nature of VR may decrease the interference of potential external distractors, which could influence the overall adaptation process and its outcomes. Further, Bourgeois et al. (2021) found that VPA progressively induce the mismatch between hand and target across trials, which reduces conscious perception of any visual bias. This progressive adaptation limits the use of conscious strategies by the subjects, potentially leading to more robust and reliable experimental outcomes (Michel et al., 2007). In summary, current research on VPA in healthy individuals is promising, demonstrating its effectiveness in inducing adaptation processes similar to PA. Moreover, there appears to be consistency in the results across studies. However, there is an urgent need to standardize methodologies and deepen our understanding of the mechanisms that produce reliable transfer effects, as well as to confirm the practical and clinical applicability of this technology. Additionally, future research should focus on assessing the ecological validity of this adaptation methodology in everyday contexts. Addressing these aspects will greatly contribute to refining VPA research and maximizing its potential clinical value.

## 5 Conclusion

Overall, this review emphasizes the potential of VPA and suggests that further investigations of its effects may support its application as possible rehabilitation approaches. VPA offers several advantages over traditional prism goggles, overcoming well known limitations. Specifically, VPA enables a gradual increase in deviations to produce greater aftereffects, reduces distraction from external stimuli, and it is suitable for bedridden patients.

Finally, there is a notable gap in studies investigating VPA in those neurological populations that may benefit from PA therapy, such as neglect patients. Understanding the duration and generalization of aftereffects to daily activities in these populations is essential. On the other hand, investigating the effects of VPA in healthy individuals represents a crucial first step paving the way for a more reliable and precise application of this innovative technology in patients, aiming to maximize benefits and reduce risks.

## Author contributions

LC: Conceptualization, Writing – original draft. AG: Writing – review & editing. VL: Writing – review & editing. VC: Writing – review & editing. AF: Supervision, Visualization, Writing – review & editing. CV: Supervision, Visualization, Writing – review & editing. AQ: Supervision, Visualization, Writing – review & editing. SM: Supervision, Visualization, Writing – review & editing.
